# Nomogram incorporating potent inflammatory indicators for overall survival estimation of patients with primary oral squamous cell carcinoma

**DOI:** 10.3389/fonc.2023.1197049

**Published:** 2023-07-14

**Authors:** Hai-xuan Wu, Shi Cheng, Fan Liu, Jun-jie Lin, Su-na Huang, Cheng-li Wang, Bin Zhou, Zhong-qi Liu, Ming-hui Cao

**Affiliations:** ^1^Department of Anesthesiology, Sun Yat-sen Memorial Hospital, Sun Yat-sen University, Guangzhou, China; ^2^Medical Research Center of Shenshan medical center, Memorial Hospital of Sun Yat-Sen University, Shanwei, China; ^3^Department of Oral and Maxillofacial Surgery, Sun Yat-sen Memorial Hospital, Sun Yat-sen University, Guangzhou, China

**Keywords:** nomogram, oral squamous cell carcinoma, prognosis, systemic inflammatory marker, lymphocyte-monocyte ratio

## Abstract

**Background:**

Inflammation has been recognized to be a factor that substantially influences tumorigenesis and tumor prognosis. Hence, this study was aimed to investigate an inflammatory marker with the most potent prognostic ability and to evaluate the survival estimation capability of dynamic change in this marker for patients suffered from oral squamous cell carcinoma (OSCC).

**Methods:**

469 patients’ inflammatory indicators including lymphocyte-to-monocyte ratio (LMR), neutrophil-to-lymphocyte ratio (NLR), platelet-to-lymphocyte ratio (PLR) and systemic inflammatory response index (SIRI), were calculated. Their predictive abilities for overall survival (OS) were evaluated by Kaplan-Meier curves to screen for the one with the most potent prognostic value. The predictive ability of dynamic changes in this marker was verified and a predictive nomogram incorporating inflammatory indicators was developed.

**Results:**

A high LMR was identified to be an indicator of a satisfactory survival rate. Compared with that of other inflammatory markers, area under the receiver operating characteristics (ROC) curve (AUC) of LMR for 1-year and 3-year OS was significantly larger (P<0.001). Dynamic LMR change remained an significant parameter for predicting OS (OR: 2.492, 95% CI: 1.246–4.981, p = 0.010). The nomogram incorporating LMR exhibited a superior prognostic significance than the TNM system, as suggested by the C-index (0.776 vs 0.651 in primary cohort; 0.800 vs 0.707 in validation cohort, P<0.001) and AUC.

**Conclusions:**

LMR was demonstrated to possess a more potent survival estimation capability than the other three inflammatory parameters. Dynamic changes in LMR serves as a significant parameter for overall survival estimation of primary OSCC patients. The established nomogram incorporating inflammatory markers showed more accuracy and sensitivity for survival estimation of primary OSCC patients.

## 1 Introduction

Oral cavity cancer, the majority of which is histologically diagnosed as squamous cell carcinoma, is the sixth leading malignancy worldwide with an incidence of approximately 744,994 new cases per year (1). Despite the availability of multimodal treatment, patients with OSCC, even in the early stage, are prone to suffer from local recurrence or metastasis after primary tumor removal (2), making its average 5-year OS rate only approximately 60% worldwide (3). Therefore, early risk stratification and spotting poor-prognosis population after standard treatment is urgently required to provide appropriate treatment.

Traditionally, clinicians evaluated the clinical outcome of OSCC patients on the grounds of the American Committee on Cancer (AJCC) tumor-node-metastasis (TNM) staging system (4). However, TNM system merely focuses on static tumor-specific features at the time of diagnosis without accounting for dynamic changes in host characteristics. Furthermore, given the individual differences, it stands to reason that the host characteristics vary from one patient to another (5, 6) and even from the preoperative to the postoperative period. Therefore, the accuracy of the TNM system is compromised in prognosis evaluation and guiding treatment decision-making for individuals. It is requisite to develop a all-sided and handy tool for survival estimation of OSCC patients.

Inflammation serves as an essential host feature and has proven to be the seventh hallmark of cancer in the last decade (7, 8). Abundant and compelling evidence has demonstrated that inflammation facilitates angiogenesis, tumor invasion and metastasis by supplying bioactive molecules to the tumor environment (9). The cancer-associated systemic inflammatory response has become a critical indicator of tumor progression (10–12). Subsequently, biomarkers reflecting the level of systemic inflammation blossom, producing a wide range of circulating blood cell count-based inflammatory markers that have helped refine prognosis prediction when incorporated with TNM system in multiple malignancies (13, 14). Nevertheless, studies investigating the optimal inflammatory biomarkers available from routine blood examination to predict clinical outcomes in OSCC patients remain limited. In addition, the reported studies were mainly conducted to analyze the prognostic capacity of inflammation markers either before or after treatment. A dearth of studies have explored the prognostic capacity of dynamic inflammatory change before and after initial treatment, which can precisely reflect therapeutic response and the change in immune surveillance condition due to changed tumor burden.

Hence, the target of our work was to investigate the inflammatory indicator with the most potent prognostic capacity and to examine its prognostic value for primary OSCC patients who underwent tumor resection and free flap reconstruction surgery. Furthermore, a prognostic nomogram incorporating inflammatory markers and clinicopathological features was developed and compared with TNM system in predicting accuracy to provide an useful tool to work out a better-individualized therapy for patients with poor prognoses.

## 2 Subjects and methods

### 2.1 Patients

789 OSCC patients underwent primary tumor ablation and free flap transplant surgery at Sun Yat-Sen Memorial Hospital between July 2017 and December 2019. Of these, 469 patients were eligible for the study and 320 were excluded based on the following criteria: 1) patients with recurrent OSCC; 2) patients who previously or concurrently diagnosed with other malignant cancers; 3) patients who diagnosed with distant metastasis; 4) patients who previously underwent anticancer therapies (radiotherapy, chemotherapy, or immunotherapy); 5) patients with postoperative infection or with diseases or a history of medication that might have an impact on the complete cell counts (systemic autoimmune disease, hematological disease, long-term steroidal treatment, etc.); 6) patients with incomplete data. A random split-sample method was used to divide 469 patients into primary and validation cohorts. The primary cohort (n=328) was used to extinguish the inflammatory biomarker with the most potent predictive value and to develop a predictive model. We verified the results that obtained from primary cohort in validation cohort. The study was approved by Sun Yat-Sen Memorial Hospital’s Medical Ethics Committee.

### 2.2 Variable collection

The following demographic data were collected: age, sex, BMI, American Society of Anesthesiology (ASA) status and comorbidities. Clinicopathological data, including TNM stage (AJCC, eighth), tumor differentiation, preoperative complete blood cell counts (CBCs) within three days before surgery and CBCs on discharge, were also collected for all patients. Length of stay (LOS) after surgery and operative variables that may have an impact on the prognosis, including duration of surgery, flap type, blood loss and intraoperative blood transfusion, were also recorded.

### 2.3 Calculation of systemic inflammatory markers based on blood cell ratios

The counts of lymphocytes, monocytes, neutrophils, and platelets in blood samples 1-3 days before surgery and on the day of discharge were collected. The calculation of systemic inflammatory markers were performed as follows. LMR was calculated as lymphocyte count/monocyte count, NLR neutrophil count/lymphocyte count, and PLR platelet count/lymphocyte count. SIRI was calculated as neutrophil count x monocyte count/lymphocyte count (10^9^/L). The appropriate truncation points of inflammatory indexes were calculated by X-Tile software (Yale University, New Haven). The dynamic change in LMR was obtained as [(LMR on discharge - LMR before surgery)/LMR before surgery]. Systemic inflammatory markers within three days before surgery were used to reflect the patient’s preoperative inflammation state, and systemic inflammatory markers on discharge were used to reflect the patient’s postoperative inflammation state. According to the definition of these inflammatory markers, LMR is proportional to lymphocyte count and inversely proportional to monocyte count, while the other three markers are opposite to it. Therefore, LMR and the other three markers would exhibit reverse trend after the surgery.

### 2.4 Follow-up

The follow-up was carried out by regular vist to oral and maxillofacial clinic and telephone calls. In the first year after discharge, we followed up the patients every 3 months and every 6 months afterwards. The follow-up was conducted until the patient’s death or the end of the study, dated May 31, 2021. Overall survival, the endpoint of interest, was calculated as the interval from operation to death or last follow-up.

### 2.5 Nomogram construction and validation

The method to establish and validate nomogram was the same as in our previous study (15). Briefly, we constructed a nomogram including the independent variables of OS which was screened by backward stepwise regression analysis. Calibration plots were developed to assess the survival discriminative capability of the nomogram for OSCC patients. For internal validation, the nomogram was fitted repeatedly in 1000 bootstrap samples to calculate the concordance index (C-index).

### 2.6 Statistical analysis

Quantitative variables following normal distribution were compard by the Student’s t-test otherwise by the Mann-Whitney U test, whereas Categorical variables were compared using χ2 or Fisher’s exact test. The OS curves were plotted graphically using the Kaplan-Meier survival analysis, and the results of subgroups that were divided by the appropriate truncation values of inflammatory parameters were compared. Univariable and multivariable analyses were applied to spot the independent risk parameters for overall survival. Then, a nomogram was generated in the primary cohort based on variables statistically significant in backward stepwise cox regression analysis. The nomogram was developed and validated by *rms* package in R. The increment in survival estimation capacity of the constructed nomogram compared with TNM system was quantified by AUC and concordance index.

## 3 Result

### 3.1 Cohort demographics

A total of 469 OSCC patients were included in the present research. The demographic data were seen in **Table 1**. Patients were assigned into primary cohort (n=328) and validation cohort (n=141) at random. In primary cohort, there were 210 males (64%) and 118 females (36%). The pathological grades of most patients were moderately differentiated (47.9%). On the basis of 8^th^ edition AJCC TNM staging system, predominant patients were in stage III (50%). Half of the tumor were located in oral tongue (51.9%). The validation cohort comprised 93 males (66%) and 48 females (34%). There were 50 well-differentiated cases (35.5%), 75 moderately differentiated cases (53.2%) and 16 poorly differentiated cases (11.3%). Nearly half patients (46.1%) were in stage III. 59.6% of cases occured in the oral tongue. The median LOS in the primary and the validation cohorts was 12 (9, 15) days and 12 (10, 14) days, respectively. No significant difference in the clinicopathological characteristics was found between these two cohorts.

**Table 1:** Clinicopathological characteristics of patients in the primary and validation cohorts.

	Primary Cohort (n=328)	Validation Cohort (n=141)	P value
Sex (male), No. (%)	210 (64.0)	93 (66.0)	0.688
Age, mean (SD), yr	56.50 (12.86)	56.52 (12.56)	0.989
BMI, mean (SD), kg/m2	22.53 (3.54)	22.53 (3.38)	0.998
Comorbidities, No. (%)			
hypertension	70 (21.3)	24 (17.0)	0.284
diabetes mellitus	35 (10.7)	13 (9.2)	0.635
stroke	3 (0.9)	4 (2.8)	0.205
coronary heart disease	17 (5.2)	7 (5.0)	0.922
other	24 (7.3)	6 (4.3)	0.214
ASA Status, No. (%)			0.975
I/II	160 (48.8)	69 (48.9)	
III/IV	168 (51.2)	72 (51.1)	
Flap Types, No. (%)			0.548
fibular flap	56 (17.1)	26 (18.4)	
anterolateral thigh flap	172 (52.4)	73 (51.8)	
posterior tibial artery flap	64 (19.5)	32 (22.7)	
radial forearm flap	36 (11.0)	10 (7.1)	
Tumor location, No. (%)			0.188
oral tongue	169 (51.5)	84 (59.6)	
gums	45 (13.7)	17 (12.1)	
floor of mouth	48 (14.6)	15 (10.6)	
buccal mucosa	39 (11.9)	20 (14.2)	
hard plate	27 (8.2)	5 (3.5)	
Histological Grade, No. (%)			0.566
well differentiated	131 (39.9)	50 (35.5)	
moderately differentiated	157 (47.9)	75 (53.2)	
poorly differentiated	40 (12.2)	16 (11.3)	
TNM stage, No. (%)			0.648
I	77 (23.5)	33 (23.4)	
II	87 (26.5)	43 (30.5)	
III	164 (50.0)	65 (46.1)	
Blood Loss, median (quartiles), ml	300 (200, 400)	300 (250, 400)	0.75
Duration of Surgery, median (quartiles), min	400 (320, 480)	400 (305, 480)	0.912
Intraoperative RBC Transfusion, No. (%)	82 (25.0)	43 (30.5)	0.217
LOS, median (quartiles),day	12(9,15)	12(10,14)	0.162

BMI, Body Mass Index; ASA, American Society of Anesthesiologists; LOS, length of stay.

### 3.2 Comparison of survival estimation ability of different inflammatory markers

The median OS was 26 (range, 18 to 36 months). 1-year and 3-year OS rates were 85% and 54%, respectively. For preoperative LMR, NLR, PLR and SIRI, the appropriate truncation values were 3.76, 2.37, 164.90 and 1.20, respectively. For postoperative LMR, NLR, PLR and SIRI, the appropriate truncation values were 1.71, 6.58, 195.00 and 4.05, respectively. Consequently, these two cohorts were separated into two sub-groups by the truncation values.

**Figure 1:**
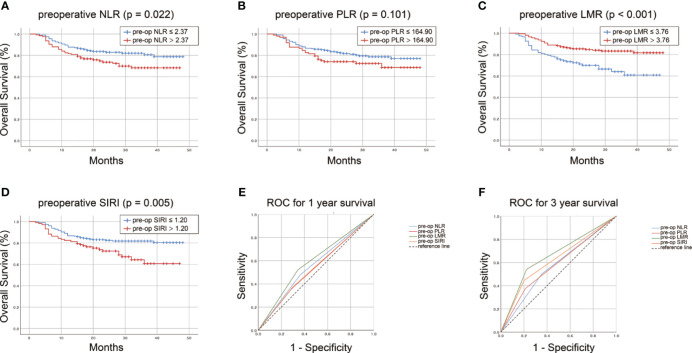
Survvial estimation capability of preoperative NLR **(A)**, PLR **(B)**, LMR **(C)**, SIRI **(D)** in the primary cohort. The survival estimation capacity of these preoperative inflammatory biomarkers was compared by AUC for 1-year **(E)** and 3-year OS **(F)**.

Kaplan-Meier curve for overall survival demonstrated a survival benefit for lower preoperative NLR (P=0.022, **Figure 1A**). No significant difference of OS rate was observed between higher and lower preoperative PLR groups (P=0.101, **Figure 1B**). A survival benefit was observed in higher preoperative LMR group (P<0.001, **Figure 1C**) and lower SIRI group (P=0.005, **Figure 1D**). Specifically, in the primary cohort, OSCC patients who in the subgroup of preoperative LMR > 3.76 enjoyed a longer survival period than those in the subgroup of preoperative LMR < 3.76 (30 months vs 21 months, P < 0.001) (**Figure 1C**). Similarly, OSCC patients who had a postoperative LMR > 1.71 underwent significantly longer survival time than those in low postoperative LMR subgroup (30 months vs 23 months, P < 0.001) (**Figure S1C**). The same tendency was found in the comparison of OS between two subgroups of NLR and SIRI (P < 0.05), but not seen in comparison of OS between the subgroups of PLR (P>0.05).

Furthermore, the predictability of these inflammatory indexes was compared by AUC. The AUC for preoperative LMR, NLR, PLR and SIRI was 0.589, 0.557, 0.534 and 0.538, respectively, for 1-year survival (**Figure 1E**) and 0.659, 0.574, 0.582 and 0.622, respectively, for 3-year survival (**Figure 1F**). Compared with that of preoperative NLR, PLR and SIRI, the AUC of preoperative LMR for 1- and 3-year OS of OSCC patients was larger (P<0.001). For postoperative LMR, NLR, PLR and SIRI, the AUC was 0.593, 0.544, 0.533 and 0.558, respectively, for 1-year survival (**Figure S1E**) and 0.668, 0.586, 0.520 and 0.629, respectively, for 3-year survival (**Figure S1F**). The AUC of the postoperative LMR was also drastically larger than that of the other postoperative indexes (P<0.001). Taken together, these data suggested that LMR possesses a superior prognostic significance for OSCC patients than the other three indexes.

We then assessed whether LMR had any potential linkage with clinicopathological characteristics. In primary cohort, the preoperative LMR was significantly associated with TNM stage (P<0.05). However, postoperative LMR had no statistical relationship with TNM staging (P>0.05, **Table S1**).

Subsequently, the 1- and 3-year OS in accordance with the truncation values of preoperative LMR and dynamic change of LMR was analyzed in **Table 2**. The result showed that the patients in subgroup of low preoperative LMR bear significantly lower 1- and 3-year survival rates than those in subgroup of high preoperative LMR (79.3% and 32.8% vs 88.9% and 66.7%, P<0.05). Likewise, OS in high and low dynamic LMR change subgroup was also statistically different. The 1- and 3-year OS were 74.4% and 37.0% in the low dynamic LMR change subgroup, 86.7% and 58.5% in high dynamic LMR change subgroup.

**Table 2:** Survival outcomes in accordance with the optimal truncation value of preoperative LMR and dynamic LMR change.

	No. of patients	OS (%)	OR	95% CI	P value
1-year					
Preoperative LMR					
**¾ 3.76**	121	79.3	2.083	1.123 to 3.864	0.020^*^
**> 3.76**	207	88.9	1		
Dynamic LMR change					
**¾ -0.76**	43	74.4	2.234	1.039 to 4.804	0.040^*^
**> -0.76**	285	86.7	1		
3-year					
Preoperative LMR					
**¾ 3.76**	58	32.8	4.105	2.060 to 8.180	<0.001^*^
**> 3.76**	99	66.7			
Dynamic LMR change					
**¾ -0.76**	27	37.0	2.393	1.017 to 5.628	0.046^*^
**> -0.76**	130	58.5	1		

^*^represents statistical significance.

LMR, lymphocyte-to-monocyte ratio; OS, overall survival; OR, Odd Ratio; CI, confidence interval.

### 3.3 Determination of independent predictive factors for prognosis

Univariate and multivariate analysis screened out potentially independent predictive parameters. All statistically significant variables found in the univariate analysis were incorporated as covariates in multivariate analyses. In primary cohort, the univariate analysis showed that age, hypertension, stroke, histological grade, TNM stage, preoperative albumin, preoperative LMR, postoperative LMR, dynamic LMR change and intraoperative RBC transfusion were significant prognostic factors. In multivariate analyses, dynamic LMR change remained an independent predictive variable of OS (OR: 2.492, 95% CI: 1.246–4.981, p = 0.010, **Table S2**). Hypertension, histological grade, TNM stage, preoperative LMR and intraoperative blood transfusion were also identified to be independent predictive variable of OS.

### 3.4 Development of the nomogram and comparison of prognostic efficacy

**Figure 2:**
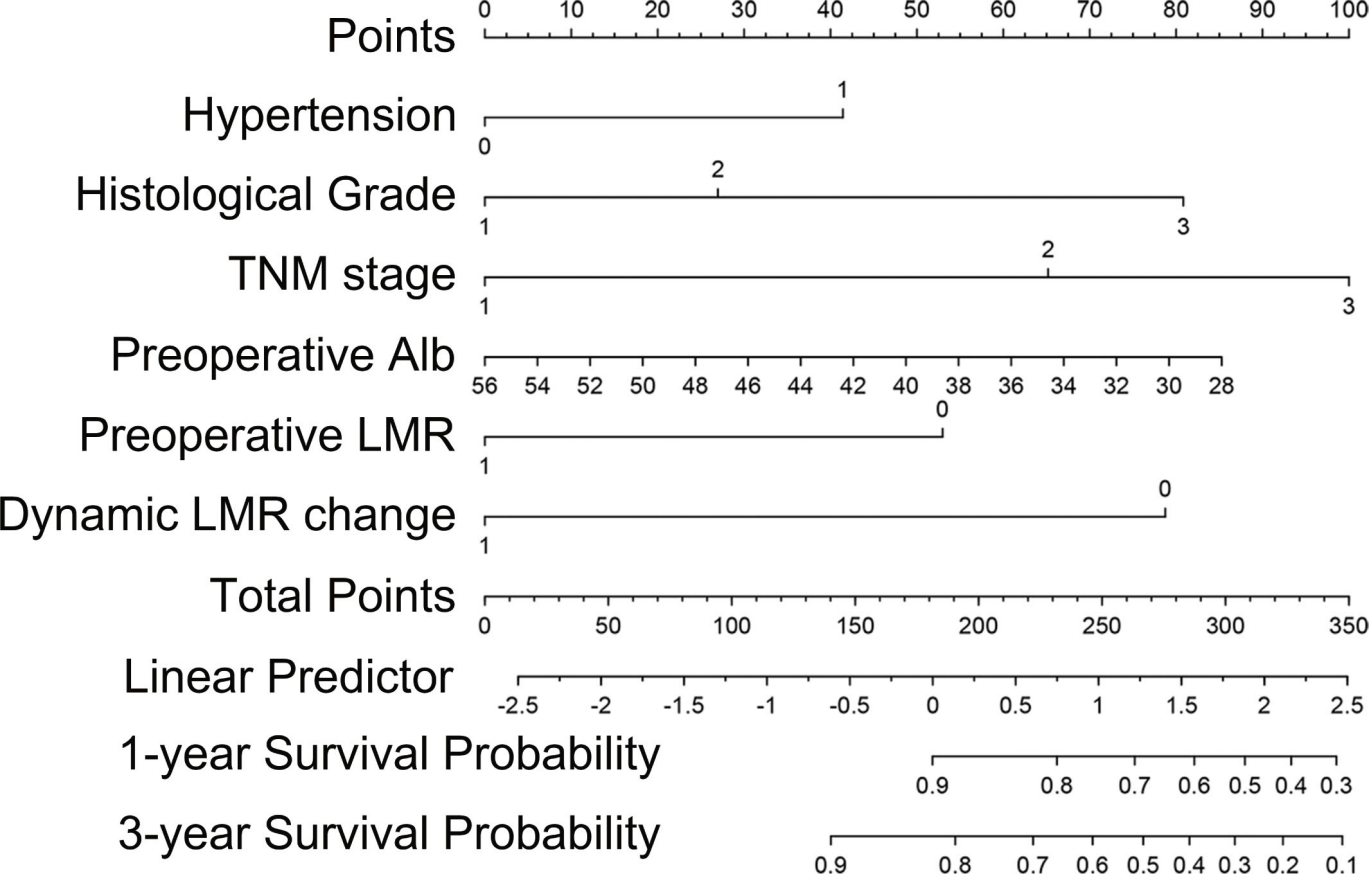
Nomogram for prognosis prediction in primary OSCC patients who underwent surgical resection and free flap reconstruction.

Backward stepwise cox regression identified hypertension, histological grade, TNM stage, preoperative albumin, preoperative LMR and dynamic LMR change as independent predictors for survival estimation. These identified parameters were incorporated to construct a predictive nomogram (**Figure 2**). The corresponding points for each parameters could be got by drawing a vertical line from the parameter axis to the points axis. After summing up all points, we can obtained the 1-year or 3-year survival probability by locating the position in the bottom axis which was right below the position of the total points in points axis.

**Figure 3:**
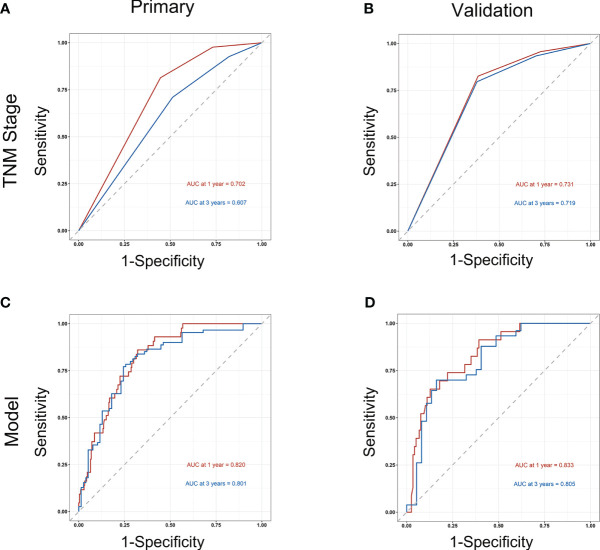
Comparison of the prognostic capacity of TNM stage and developed nomogram by 1- and 3-year ROC curves in primary cohort **(A, C)** and in validation cohort **(B, D)**.

**Figure 4:**
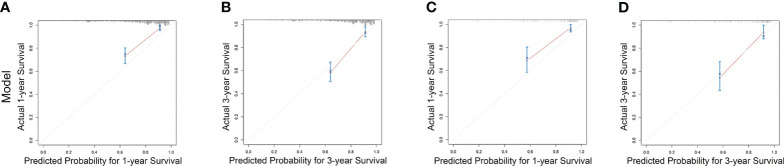
Calibration curves for nomogram estimating 1- and 3-year overall survival rates in primary cohort **(A, B)** and validation cohort **(C, D)**.

Subsequently, survival estimation capacity of developed nomogram was compared with that of TNM system. In primary cohort, the concordance index indicated that the developed nomogram holds a better prognostic ability for overall survival than TNM system (0.776, 95% CI: 0.729-0.823 vs 0.651 95% CI: 0.602-0.700, **Table 3**). With regard to ROC curve, nomogram presented larger area under ROC curve than TNM system for predicting 1- and 3-year overall survival (0.820, 95% CI: 0.765-0.875 vs 0.702, 95% CI: 0.645-0.760 (1-year overall survival); 0.801, 95% CI 0.728-0.874 vs 0.607, 95% CI 0.528-0.686 (3-year overall survival), **Figure 3**). In validation cohort, the concordance index of developed nomogram was 0.800 (95% CI: 0.733–0.867), which was dramatically higher than that of TNM system (0.707, 95% CI: 0.640–0.774, **Table 3**). Likewise, AUC for developed nomogram was also bigger than that of TNM system (0.833, 95% CI: 0.752-0.914 vs 0.731, 95% CI: 0.645-0.817(1-year OS); 0.805, 95% CI: 0.702-0.908 vs 0.719, 95% CI: 0.613-0.825 (3-year OS), **Figure 3**). These results indicated that the nomogram integrating the clinicopathological variables and inflammatory biomarkers was a superior method for the OS prediction of OSCC patients. Lastly, the calibration curve of nomogram was presented graphically to determine the matching degree of actual observation rates and nomogram-predicted rates. As shown in **Figure 4**, an excellent match was observed, indicating the reliable predicting ability of the established nomogram for overall survival of OSCC patients.

**Table 3:** Risk factors for OS derived from backward stepwise cox regression analyses.

		P value	HR	95% CI
**Hypertension**		0.021^*^	0.545	0.326 to 0.911
**Histological Grade, No. (%)**		0.001^*^		
well differentiated		<0.001^*^	0.306	0.157 to 0.595
moderately differentiated		0.007^*^	0.457	0.259 to 0.806
poorly differentiated		NA		
**TNM stage, No. (%)**		0.004^*^		
I		0.002^*^	0.228	0.090 to 0.581
II		0.082	0.602	0.339 to 1.067
III		NA		
Preoperative				
albumin		0.112	0.955	0.903 to 1.011
LMR		0.005^*^	2.190	1.264 to 3.794
**Dynamic LMR change**		<0.001^*^	3.216	1.714 to 6.035
**C-index of TNM**	**Primary**		0.651	0.602 to 0.700
	**Validation**		0.707	0.640 to 0.774
**C-index of nomogram**	**Primary**		0.776	0.729 to 0.823
	**Validation**		0.800	0.733 to 0.867

^*^represents statistical significance.

LMR, lymphocyte-to-monocyte ratio; HR, Hazard ratio; CI, confidence interval; NA, not available.

## 4 Discussion

In present study, we investigated the survival estimation capacity of inflammatory parameters LMR, NLR, PLR and SIRI for primary OSCC patients who underwent tumor resection and free flap reconstruction. The results indicated that the prognostic abilities of both preoperative and postoperative LMR were better than those of the other systemic inflammatory biomarkers. A lower LMR was associated with unsatisfactory prognosis. Notably, it was demonstrated that the dynamic change in LMR was an independent indicator for the OS of primary OSCC patients. Specifically, the patients with dynamic change of LMR<-0.76 underwent lower OS than patients with dynamic change of LMR>-0.76. If the postoperiatve LMR of the patient was lower, the dynamic change of LMR of this patient was more likely to lower than -0.76, which is correlated with unsatisfactory prognosis. These results exhibited cosistentcy suggesting that the inflammatory marker LMR and the dynamic change of LMR could be used to refine prognostic stratification. For all we know, our work is the first study evaluating both preoperative and postoperative inflammatory biomarkers to discriminate the one that possesses the most potent prognostic value for primary OSCC patients and is also the first study investigating the survival estimation ability of dynamic changes in LMR in primary OSCC. Further, we established and validated a nomogram integrating clinicopathological features, preoperative LMR and dynamic changes in LMR that outperformed the traditional TNM system in terms of prognostic value. The proposed nomogram can provide valuable survival prediction information to refine the decision-making process for OSCC patients.

Previous studies on the association between oral cancer prognosis and systemic inflammation have shown that systemic inflammation in the host correlates with the genesis and progression of oral cancer (16, 17). Particularly, systemic inflammation can stimulate the advancement, metastasis and recurrence of oral cancer, which closely relates to the survival rate of cancer patients (16, 18). From the perspecitve of many cancer researchers, the systemic inflammation could be viewed as an interaction between tumor-derived components released into the blood stream and the immune system at the systemic level. Once the tumor is removed, the different tumor components released into circulation would reduce dramatically and the interaction will be interrupted (19), which would lead to the change in inflammation and immune state. Does this varied inflammatory and immune state of host lead to change in prognosis? If the prognosis would change, then, compared with preoperative inflammatory level, what level should postoperative inflammation be reduced to so that the survival rates of OSCC patients will improve? The answers to none of these questions are yet clear. Given this, we first screened for the optimal inflammatory biomarker of prognostic significance. Then, its dynamic change before and after initial treatment was calculated to explore the prognostic ability of the change in inflammatory state. According to our results, the survival estimation capacity of LMR is more superior than that of the other three indexes assessed in the research, and a low LMR is an indicator of poor prognosis. Moreover, dynamic change in LMR was proved to be an independent prognostic parameter for overall survival of OSCC patients and dynamic change of LMR>-0.76 was proved to be correlated with better prognosis, suggesting that the inflammatory marker could vary with changes in tumor load and precisely reflect the tendency of tumor progression.

To date, the mechanism of a low LMR giving rise to an compromised prognosis in OSCC has not been elucidated. According to molecular-based studies in recent decades, peripheral blood cells are increasingly recognized as regulators of tumor progression and metastasis beyond their crucial roles in clearing exogenous antigens and maintaining hemostasis. Chronic inflammation characterized by persistently elevated leukocytes is termed tumorigenic chronic inflammation, which is an intertwined process with cancer progression (20, 21). One population of the main effector cells participating in the progression of inflammatory response is monocyte, which regulate inflammation state through the synthesis of cytokines and chemokines and regulation of lymphocyte activation. Monocytes in peripheral blood can be recruited chemotactically to the site of inflammation or tumor microenvironment and subsequently be educated to obtain a protumoral phenotype characterized by secreting abundant proinflammatory cytokines to promote the inflammatory tumor microenvironment (22). Other mechanisms have been published emphasizing monocyte’s role in tumor-promoting activity through i) abundantly expressing SIRP-α, PD-L1 and other immune-regulatory receptors that suppress the antitumor immune response, ii) priming the premetastatic site, and iii) promoting tumor cell metastasis by producing matrix metalloproteinase and regulating epithelial-mesenchymal transition (23–25). In contrast, lymphocytes have been well characterized for their antitumor effect and have a great impact in the immunologic killing of malignant cells (26). Notably, CD8+ T cytotoxic lymphocytes mediate direct killing of target tumor cells by producing cytotoxins such as perforin and granzyme and through the Fas-FasL pathway. A low lymphocyte count serves as an unsatisfactory prognosis indicator in patients suffered from malignancies (27, 28). LMR is an inflammatory biomarker based on these two blood cell counts, which will be minimized when a decreased lymphocyte count and an increased monocyte count are present. Thus, LMR could be a surrogate for the balance between tumor-promoting inflammation and antitumor immune surveillance and can be applied to the prognosis prediction of OSCC patients.

Recently, there has been an increasing trend to develop nomogram for cancer prognosis, primarily because of their user-friendly graphical interfaces enabling visualization of the prognostic strength of various relevant factors (29, 30). Compared with the traditional TNM system, the advantage of nomogram is its ability to incorporate a wide array of prognostic indicators to formulate a noninvasive tool that could predict the prognosis according to an individual patient’s profile (31, 32). In our work, we constructed a predictive nomogram on the basis of six independent prognostic parameters (hypertension, histological grade, TNM stage, preoperative albumin, preoperative LMR and dynamic change in LMR) identified by the backward stepwise cox regression analyses. The nomogram exhibited more sensitivity and specificity than the conventional TNM staging system in estimating 1- and 3-year OS probability. The potent survival estimation capability of constructed nomogram was also demonstrated by the higher C-index and the fact that its calibration curve was closely matched to the ideal standard line.

There are two main limitations to our study. First, due to the nature of the retrospective design, the selection bias was inevitable. Second, patients diagnosed with distant metastasis and with recurrent OSCC were systematically excluded from our study. This means that our results cannot be generalized to patients with these characteristics. External validation in heterogeneous cohorts or a prospective study is imperative for the extrapolation of our study.

## 5 Conclusions

We found that the inflammatory biomarker LMR possesses a more potent prognostic value than NLR, PLR and SIRI for primary OSCC patients. Furthermore, for the first time, the dynamic change in LMR was demonstrated to be an independent indicator for OS of primary OSCC patients, and a predictive nomogram including this factor was developed and validated. In comparison of the conventional TNM system, our constructed nomogram incorporating dynamic LMR showed more satisfactory prognostic value which supported the essential role of inflammation in prognosis.

## Data availability statement

The original contributions presented in the study are included in the article/**Supplementary Material**. Further inquiries can be directed to the corresponding authors.

## Ethics statement

This retrospective study protocol was approved by the Medical Ethics Committee of Sun Yat-Sen Memorial Hospital.

## Author contributions

All authors contributed to the study conception and design. Data collection and analysis were performed by H-XW, SC, FL, Z-QL, J-JL, C-LW and S-NH. BZ, Z-QL and M-HC supervised the project. Acquisition of the funding was performed by M-HC. The first draft of the manuscript was written by H-XW and all authors commented on previous versions of the manuscript All authors contributed to the article and approved the submitted version.

## Supplementary material

Supplementary Figure 1 Survvial estimation capability of postoperative NLR **(A)**, PLR **(B)**, LMR **(C)**, SIRI **(D)** in the primary cohort. The predictive ability of these postoperative inflammatory biomarkers was compared by AUC in 1-year **(E)** and 3-year OS **(F)**.

## References

[1] Sung H Ferlay J Siegel RL Laversanne M Soerjomataram I Jemalet A. Global cancer statistics 2020: GLOBOCAN estimates of incidence and mortality worldwide for 36 cancers in 185 countries. CA Cancer J Clin (2021) 71(3):209–49. doi: 10.3322/caac.21660 33538338

[2] Zhan KY Morgan PF Neskey DM Kim JJ Huang AT Garrett-Mayer E. Preoperative predictors of occult nodal disease in cT1N0 oral cavity squamous cell carcinoma: review of 2623 cases. Head Neck (2018) 40(9):1967–76. doi: 10.1002/hed.25178 29761586

[3] Siegel RL Miller KD Jemal A. Cancer statistics. CA Cancer J Clin (2020) 70(1):7–30. doi: 10.3322/caac.21590 31912902

[4] Sridharan S Thompson LDR Purgina B Sturgis CD Shah AA Burkey B. Early squamous cell carcinoma of the oral tongue with histologically benign lymph nodes: A model predicting local control and vetting of the eighth edition of the American Joint Committee on Cancer pathologic T stage. Cancer (2019) 125(18):3198–207. doi: 10.1002/cncr PMC7723468 31174238

[5] Valero C Zanoni DK Pillai A Ganly I Morris LGT Shah PJ. Host factors independently associated with prognosis in patients with oral cavity cancer. JAMA Otolaryngol Head Neck Surg (2020) 146(8):699–707. doi: 10.1001/jamaoto.2020.1019 32525545 PMC7290709

[6] Siriwardena BS Tilakaratne A Amaratunga EA Tilakaratne WM. Demographic, aetiological and survival differences of oral squamous cell carcinoma in the young and the old in Sri Lanka. Oral Oncol (2006) 42(8):831–6. doi: 10.1016/j.oraloncology.2005.12.001 16527511

[7] Hanahan D Weinberg RA. Hallmarks of cancer: the next generation. Cell (2011) 144(5):646–74. doi: 10.1016/j.cell.2011.02.013 21376230

[8] Balkwill F Mantovani A. Inflammation and cancer: back to virchow? Lancet (London England) (2001) 357(9255):539–45. doi: 10.1016/s0140-6736(00)04046-0 11229684

[9] Grivennikov SI Greten FR Karin M. Immunity, inflammation, and cancer. Cell (2010) 140(6):883–99. doi: 10.1016/j.cell.2010.01.025 PMC2866629 20303878

[10] Sherry AD von Eyben R Newman NB Gutkin P Mayer I Horst K. Systemic inflammation after radiation predicts locoregional recurrence, progression, and mortality in stage II-III triple-negative breast cancer. Int J Radiat Oncol Biol Phys (2020) 108(1):268–76. doi: 10.1016/j.ijrobp.2019.11.398 PMC7473500 31809877

[11] Wesselink E Balvers MGJ Kok DE Winkels RW Zutphen MV Schrauwen RWM. Levels of inflammation markers are associated with the risk of recurrence and all-cause mortality in patients with colorectal cancer. Cancer Epidemiol Biomarkers Prev (2021) 30(6):1089–99. doi: 10.1158/1055-9965.EPI-20-1752 33771850

[12] Yoshimura T Suzuki H Takayama H Higashi S Hirano Y Tezuka M. Prognostic value of inflammatory biomarkers in aged patients with oral squamous cell carcinoma. Front Pharmacol (2022) 13:996757. doi: 10.3389/fphar.2022.996757 36479205 PMC9719958

[13] Chen L Qian J Lin L Lin J Chen Q Zhuang C. Prognostic value of preoperative lymphocyte-to-monocyte ratio in oral cancer patients and establishment of a dynamic nomogram. Oral Dis (2021) 27(5):1127–36. doi: 10.1111/odi.13629 32881142

[14] Suzuki Y Okabayashi K Hasegawa H Tsuruta M Shigeta K Kondo T. Comparison of preoperative inflammation-based prognostic scores in patients with colorectal cancer. Ann Surg (2018) 267(3):527–31. doi: 10.1097/SLA.0000000000002115 27984214

[15] Liu ZQ Wu HX Liufu N Cheng S Huang HQ Hu CW. Development and validation of a nomogram incorporating selected systemic inflammation-based prognostic marker for complication prediction after vascularized fibular flap reconstruction. Oral Oncol (2019) 99:104467. doi: 10.1016/j.oraloncology.2019.104467 31678763

[16] Eltohami YI Kao HK Lao WW Huang YL Abdelrahman M Liao CT. The prediction value of the systemic inflammation score for oral cavity squamous cell carcinoma. Otolaryngol Head Neck Surg (2018) 158(6):1042–50. doi: 10.1177/0194599817751678 29336202

[17] Diao P Wu Y Li J Zhang W Huang R Zhou C. Preoperative systemic immune-inflammation index predicts prognosis of patients with oral squamous cell carcinoma after curative resection. J Transl Med (2018) 16(1):365. doi: 10.1186/s12967-018-1742-x 30563540 PMC6299596

[18] Feller L Altini M Lemmer J. Inflammation in the context of oral cancer. Oral Oncol (2013) 49(9):887–92. doi: 10.1016/j.oraloncology.2013.07.003 23910564

[19] Diakos CI Charles KA McMillan DC Clarke SJ. Cancer-related inflammation and treatment effectiveness. Lancet Oncol (2014) 15(11):e493–503. doi: 10.1016/S1470-2045(14)70263-3 25281468

[20] Coussens LM Zitvogel L Palucka AK. Neutralizing tumor-promoting chronic inflammation: a magic bullet? Science (2013) 339:286–91. doi: 10.1126/science.1232227 PMC3591506 23329041

[21] Zhong Z Sanchez-Lopez E Karin M. Autophagy, inflammation, and immunity: a troika governing cancer and its treatment. Cell (2016) 166:288–98. doi: 10.1016/j.cell.2016.05.051 PMC4947210 27419869

[22] Merino A Buendia P Martin-Malo A Aljama P Ramirez R Carracedo J. Senescent CD14+CD16+ monocytes exhibit proinflammatory and proatherosclerotic activity. J Immunol (2011) 186(3):1809–15. doi: 10.4049/jimmunol.1001866 21191073

[23] Qian BZ Pollard JW. Macrophage diversity enhances tumor progression and metastasis. Cell (2010) 141(1):39–51. doi: 10.1016/j.cell.2010.03.014 20371344 PMC4994190

[24] Noy R Pollard JW. Tumor-associated macrophages: from mechanisms to therapy. Immunity (2014) 41(1):49–61. doi: 10.1016/j.immuni.2014.06.010 25035953 PMC4137410

[25] Shapouri-Moghaddam A Mohammadian S Vazini H Taghadosi M Esmaeili SA Mardani F. Macrophage plasticity, polarization, and function in health and disease. J Cell Physiol (2018) 233(9):6425–40. doi: 10.1002/jcp.26429 PMC13482560 29319160

[26] Miggelbrink AM Jackson JD Lorrey SJ Srinivasan ES Waibl-Polania J Wilkinson DS. CD4 T-cell exhaustion: does it exist and what are its roles in cancer? Clin Cancer Res (2021) 27(21):5742–52. doi: 10.1158/1078-0432.CCR-21-0206 PMC8563372 34127507

[27] Cho O Oh YT Chun M Noh OK Hoe JS Kim H. Minimum absolute lymphocyte count during radiotherapy as a new prognostic factor for nasopharyngeal cancer. Head Neck (2016) 38(Suppl 1):E1061–7. doi: 10.1002/hed.24158 26040623

[28] Ray-Coquard I Cropet C Glabbeke MV Sebban C Cesne AL Judson I. Lymphopenia as a prognostic factor for overall survival in advanced carcinomas, sarcomas, and lymphomas. Cancer Res (2009) 69:5383–91. doi: 10.1158/0008-5472.CAN-08-3845 PMC2775079 19549917

[29] Lu Y Yan Y Li B Liu M Liang YC Ye YS. A novel prognostic model for oral squamous cell carcinoma: the functions and prognostic values of RNA-binding proteins. Front Oncol (2021) 11:592614. doi: 10.3389/fonc.2021.592614 34395233 PMC8362834

[30] Hu JF Song X Zhong K Zhao XK Zhou FY Xu RH. Increases prognostic value of clinical-pathological nomogram in patients with esophageal squamous cell carcinoma. Front Oncol (2023) 13:997776. doi: 10.3389/fonc.2023.997776 36865805 PMC9973522

[31] Tang LQ Li CF Li J Chen WH Chen QY Yuan Lx. Establishment and validation of prognostic nomograms for endemic nasopharyngeal carcinoma. J Natl Cancer Inst (2015) 108(1):djv291. doi: 10.1093/jnci/djv291 26467665

[32] Yang Y Zhang YJ Zhu Y Cao JZ Yuan ZY Xu LM. Prognostic nomogram for overall survival in previously untreated patients with extranodal NK/T-cell lymphoma, nasal-type: a multicenter study. Leukemia (2015) 29:1571–7. doi: 10.1038/leu.2015.44 25697894

