# Supplementary figures and images for: Nomogram incorporating potent inflammatory indicators for overall survival estimation of patients with primary oral squamous cell carcinoma
Source: Front Oncol. 2023 Jul 14;13:1197049. doi: 10.3389/fonc.2023.1197049 (PMC10376696; doi:10.3389/fonc.2023.1197049)

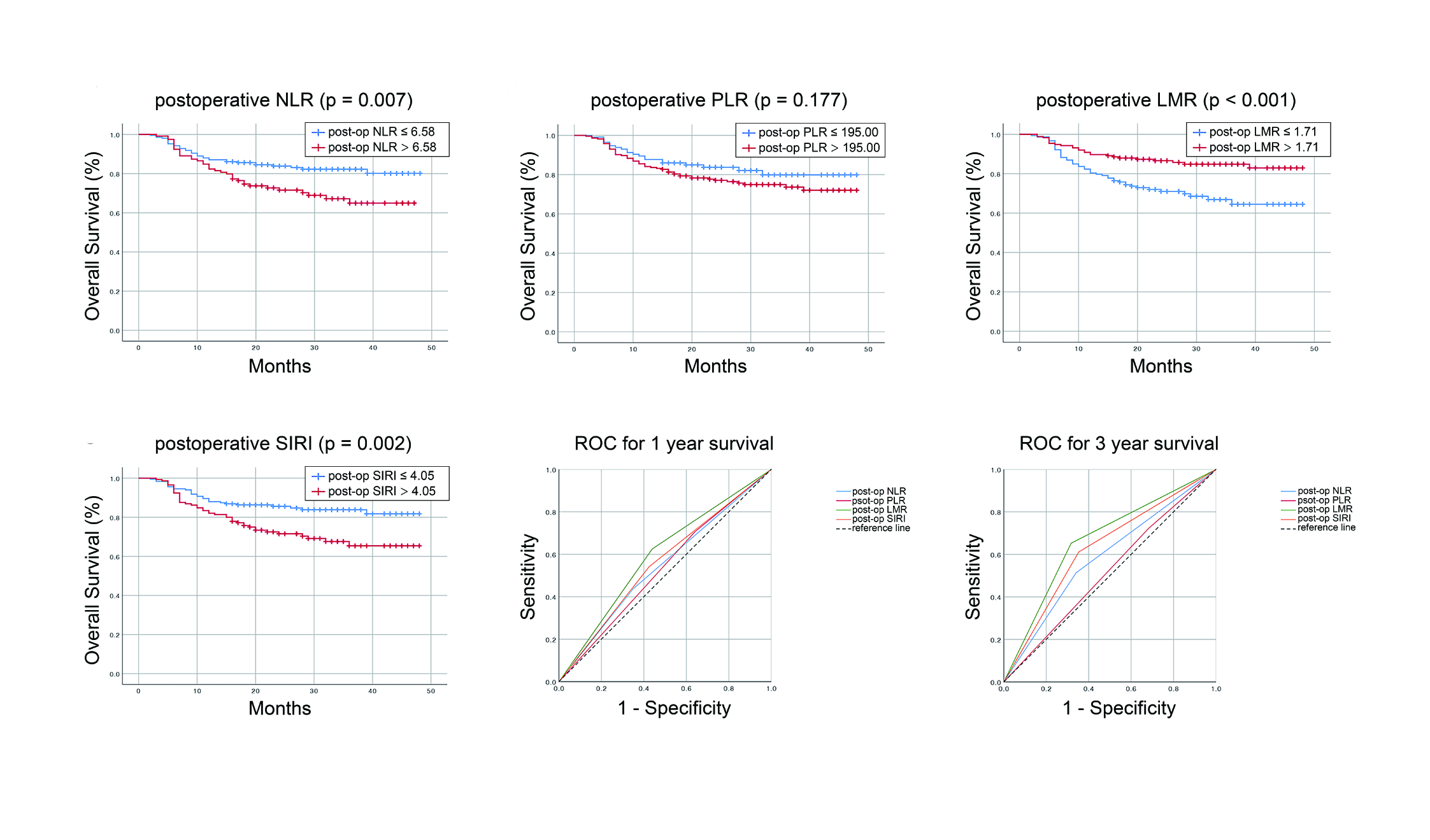

Supplement: Supplementary Figure 1 — Survvial estimation capability of postoperative NLR (A), PLR (B), LMR (C), SIRI (D) in the primary cohort. The predictive ability of these postoperative inflammatory biomarkers was compared by AUC in 1-year (E) and 3-year OS (F). [file Image_1.tif]
