# Supplementary material for: Nomogram incorporating potent inflammatory indicators for overall survival estimation of patients with primary oral squamous cell carcinoma
Source: Front Oncol. 2023 Jul 14;13:1197049. doi: 10.3389/fonc.2023.1197049 (PMC10376696; doi:10.3389/fonc.2023.1197049)
Supplement: Supplementary file 2 [file Table_1.docx]

**Table S1** Correlations between preoperative/postoperative LMR and clinicopathological characteristics

|  | **Pre-op LMR < 3.76**  **(n= 121)** | **Pre-op LMR > 3.76**  **(n=207)** | **P value** | **Post-op LMR < 1.71**  **(n= 153)** | **Post-op LMR > 1.71**  **(n=175)** | **P value** |
| --- | --- | --- | --- | --- | --- | --- |
| **Sex (male), No. (%)** | 95 (78.5) | 115 (55.6) | <0.001^*^ | 114 (74.5) | 96 (54.9) | <0.001^*^ |
| **Age, mean (SD), yr** | 60.11 (11.82) | 54.39 (13.01) | <0.001^*^ | 58.33 (12.82) | 54.90 (12.73) | 0.016^*^ |
| **Tumor location, No. (%)** |  |  | 0.505 |  |  | 0.760 |
| oral tongue | 55 (45.5) | 114 (55.1) |  | 76 (49.7) | 93 (53.1) |  |
| gums | 20 (16.5) | 25 (12.1) |  | 23 (15.0) | 22 (12.6) |  |
| floor of mouth | 19 (15.7) | 29 (14.0) |  | 20 (13.1) | 28 (16.0) |  |
| buccal mucosa | 15 (12.4) | 24 (11.6) |  | 21 (13.7) | 18 (10.3) |  |
| hard plate | 12 (9.9) | 15 (7.2) |  | 13 (8.5) | 14 (8.0) |  |
| **Histological Grade, No. (%)** |  |  | 0.308 |  |  | 0.525 |
| well differentiated | 48 (38.7) | 83 (40.1) |  | 60 (39.2) | 71 (40.6) |  |
| moderately differentiated | 54 (44.6) | 104 (49.8) |  | 71 (46.4) | 86 (49.1) |  |
| poorly differentiated | 19 (15.7) | 21 (10.1) |  | 22 (14.4) | 18 (10.3) |  |
| **TNM stage, No. (%)** |  |  | 0.040^*^ |  |  | 0.248 |
| I | 19 (15.7) | 58 (28.0) |  | 33 (21.6) | 44 (25.1) |  |
| II | 35 (28.9) | 52 (25.1) |  | 36 (23.5) | 51 (29.1) |  |
| III | 67 (55.4) | 97 (46.9) |  | 84 (54.9) | 80 (45.7) |  |

^*^represents statistical significance

**Table S2** Univariate and multivariate analyses for OS in OSCC patients

|  | **Univariate** | | | **Multivariate** | | |
| --- | --- | --- | --- | --- | --- | --- |
|  | **P value** | **OR** | **95% CI** | **P value** | **OR** | **95% CI** |
| **Sex (male)** | 0.283 | 1.315 | 0.798 to 2.169 |  |  |  |
| **Age** | 0.032^*^ | 1.021 | 1.002 to 1.040 | 0.575 | 1.006 | 0.985 to 1.027 |
| **BMI** | 0.609 | 1.02 | 0.946 to 1.099 |  |  |  |
| **Comorbidities** |  |  |  |  |  |  |
| hypertension | 0.004^*^ | 0.492 | 0.302 to 0.802 | 0.039^*^ | 0.560 | 0.323 to 0.972 |
| diabetes mellitus | 0.251 | 1.703 | 0.682 to 4.224 |  |  |  |
| stroke | 0.035^*^ | 0.22 | 0.054 to 0.899 | 0.413 | 0.513 | 0.104 to 2.533 |
| coronary heart disease | 0.169 | 0.556 | 0.241 to 1.283 |  |  |  |
| other | 0.077 | 0.533 | 0.265 to 1.071 |  |  |  |
| **ASA Status (I /II vs III/IV)** | 0.937 | 0.982 | 0.620 to 1.554 |  |  |  |
| **Flap Types** | 0.107 |  |  |  |  |  |
| fibular flap | 0.597 | 1.382 | 0.416 to 4.595 |  |  |  |
| anterolateral thigh flap | 0.065 | 2.623 | 0.941 to 7.314 |  |  |  |
| posterior tibial artery flap | 0.07 | 2.746 | 0.921 to 8.184 |  |  |  |
| radial forearm flap | NA |  |  |  |  |  |
| **Tumor location** | 0.386 |  |  |  |  |  |
| oral tongue | 0.621 | 0.911 | 0.631 to 1.317 |  |  |  |
| gums | 0.950 | 0.982 | 0.567 to 1.702 |  |  |  |
| floor of mouth | 0.296 | 0.737 | 0.416 to 1.306 |  |  |  |
| buccal mucosa | 0.681 | 0.882 | 0.484 to 1.606 |  |  |  |
| hard plate | NA |  |  |  |  |  |
| **Histological Grade, No. (%)** | <0.001^*^ |  |  | 0.001^*^ |  |  |
| well differentiated | <0.001 | 0.217 | 0.113 to 0.417 | <0.001 | 0.299 | 0.153 to 0.584 |
| moderately differentiated | 0.002 | 0.411 | 0.236 to 0.715 | 0.010 | 0.459 | 0.254 to 0.829 |
| poorly differentiated | NA |  |  | NA |  |  |
| **TNM stage, No. (%)** | <0.001^*^ |  |  | 0.003^*^ |  |  |
| I | <0.001 | 0.181 | 0.072 to 0.454 | 0.002 | 0.221 | 0.086 to 0.569 |
| II | 0.024 | 0.523 | 0.299 to 0.917 | 0.073 | 0.576 | 0.315 to 1.052 |
| III | NA |  |  | NA |  |  |
| **Preoperative** |  |  |  |  |  |  |
| hemoglobin | 0.172 | 0.99 | 0.977 to 1.004 |  |  |  |
| albumin | 0.002^*^ | 0.916 | 0.866 to 0.969 | 0.320 | 0.969 | 0.911 to 1.031 |
| LMR | <0.001^*^ | 2.316 | 1.460 to 3.675 | 0.049 | 1.824 | 1.001 to 3.323 |
| **Postoperative** |  |  |  |  |  |  |
| hemoglobin | 0.769 | 1.002 | 0.989 to 1.016 |  |  |  |
| albumin | 0.291 | 0.972 | 0.921 to 1.025 |  |  |  |
| LMR | 0.001^*^ | 0.431 | 0.267 to 0.697 | 0.101 | 1.595 | 0.913 to 2.787 |
| **Dynamic LMR change** | 0.025^*^ | 1.889 | 1.085 to 3.289 | 0.010^*^ | 2.492 | 1.246 to 4.981 |
| **Blood Loss** | 0.888 | 1 | 0.999 to 1.001 |  |  |  |
| **Duration of Surgery** | 0.928 | 1 | 0.998 to 1.002 |  |  |  |
| **Intraoperative RBC Transfusion** | 0.007^*^ | 0.522 | 0.324 to 0.839 | 0.884 | 0.959 | 0.550 to 1.674 |

^*^represents statistical significance.

Abbreviations: BMI, Body Mass Index; ASA, American Society of Anesthesiologists; LMR, lymphocyte-to-monocyte ratio; OR, Odd Ratio; CI, confidence interval, NA, not available.
